# *Magnolia figo* Extract Induces Enamel Shade Recovery and Inhibits *Porphyromonas gingivalis* Biofilm Formation: An In Vitro, Dual-Action Natural Therapeutic Approach

**DOI:** 10.3390/ijms26178157

**Published:** 2025-08-22

**Authors:** Chun-Sheng Kuo, Cheng-Wen Lin, Yuan-Man Hsu, Jen-Chieh Tsai, Dan-Jae Lin

**Affiliations:** 1 PhD Program for Health Science and Industry, China Medical University, Taichung 404328, Taiwan; u110310201@cmu.edu.tw (C.-S.K.); cwlin@mail.cmu.edu.tw (C.-W.L.); 2 Fethiann Molecule Applied Co., Ltd., Hsinchu Science Park, Yilan 260011, Taiwan; 3 Department of Medical Laboratory Science and Biotechnology, China Medical University, Taichung 404328, Taiwan; 4 Department of Animal Science and Biotechnology, Tunghai University, Taichung 407224, Taiwan; yuanmh@thu.edu.tw; 5 School of Pharmacy, China Medical University, Taichung 406040, Taiwan; jenchieh@mail.cmu.edu.tw; 6 School of Dentistry, College of Dentistry, China Medical University, Taichung 404328, Taiwan; 7 Department of Biomedical Engineering, College of Biomedical Engineering, China Medical University, Taichung 404328, Taiwan

**Keywords:** *Magnolia figo* flower extract (FMO), active phytochemical ingredient (API), enamel whitening, oral, sesquiterpenes, fourier-transform infrared spectroscopy (FTIR), scanning electron microscopy (SEM), *Porphyromonas gingivalis*, biofilm inhibition, cytocompatibility

## Abstract

Dental enamel discoloration, extrinsic staining, and periodontal biofilms remain persistent challenges in oral health. This study explores the in vitro, dual-functional potential of *Magnolia figo* flower extract (FMO), a sesquiterpene-rich botanical active phytochemical ingredient (API), for aesthetic and antimicrobial oral applications. FTIR identified characteristic terpenoid and long-chain fatty acid functional groups, including β-elemene, γ-elemene, and caryophyllene oxide. Whitening efficacy on coffee-stained bovine enamel was quantified using CIELAB colorimetry. The 0.5% FMO treatment achieved ΔE* = 8.49, which was within the clinical perceptibility threshold and the optimal biocompatibility balance. SEM confirmed no demineralization on the enamel surface after immersion in 3.0% FMO for 12 h. Antimicrobial assays demonstrated inhibition of *Porphyromonas gingivalis*, with MIC and MBC values of 0.25% and 0.5%, respectively. Biofilm formation was reduced by over 50% at a 0.148% concentration. Cytocompatibility assays using HGF-1 cells with various concentrations of FMO showed reduced cell viability at higher concentrations. When exposed for 5 min (simulating daily oral care) or 2 h, 0.5% FMO exhibited greater biocompatibility with L929 cells compared to toothpaste and peroxide-based agents. These findings suggest that FMO may serve as a natural candidate for dual-function oral care; however, further in vivo and clinical investigations are needed to validate its potential use within oral care treatments.

## 1. Introduction

Dental care products are increasingly modulated by consumer demand for whitening, stain removal, and periodontitis prevention [1,2], highlighting the significant intersection of health and economy. These demands reflect a global trend in which oral health is recognized as integral to systemic health and thus is a rapidly expanding component of the healthcare market. However, in an era of heightened health awareness, concerns have been raised regarding the potential adverse effects of conventional synthetic bleaching agents such as hydrogen peroxide and carbamide peroxide [3,4]. These compounds, while effective, have been associated with enamel sensitivity, gingival irritation, and, in some cases, long-term enamel demineralization [3,4,5]. For instance, fluoride-containing home-use whitening kits are commonly linked to temperature-induced hypersensitivity [5] and microstructural changes in enamel [6]. Dental enamel, composed primarily of hydroxyapatite (Ca_10_(PO_4_)_6_(OH)_2_), is one of the most mineralized tissues in the human body. However, it remains highly susceptible to extrinsic and intrinsic staining, acidic erosion, and microbial colonization [7,8,9]. Although peroxide-based whitening is considered a clinical gold standard, prolonged or unsupervised use has been reported to cause enamel degradation at the microscopic level, with demineralization depths reaching up to 250 μm [3,10].

Meanwhile, periodontal disease—primarily caused by pathogenic bacterial biofilms such as *P. gingivalis*—remains one of the most prevalent oral health issues globally, particularly in aging populations [11,12,13]. Emerging studies have reported potential associations between periodontal pathogens such as *P. gingivalis* and systemic health conditions, including neurodegenerative and cardiovascular diseases [13,14]. Recent studies have confirmed the presence of *P. gingivalis* in the brains of Alzheimer’s patients [15], and several inflammatory biomarkers, including CRP, serum amyloid, TNF-α, interleukin IL-6, IL-8, IL-17A, and calprotectin, are significantly increased in inflammatory bowel disease patients [14]. However, these systemic implications remain outside the scope of the present study and are referenced here to underscore the broader significance of periodontal biofilm control.

To address these dual oral health challenges—extrinsic enamel discoloration and periodontal infection—plant-based alternatives have gained increasing scientific attention. Botanical formulations have demonstrated potential as safer adjuncts in oral care due to their whitening, antimicrobial, and anti-inflammatory properties, and are already being incorporated into herbal dentifrices and mouthwashes, as supported by an increasing body of scientific literature over the past decade [16,17,18,19]. Despite the increasing availability of such natural ingredients, only a limited number of studies have rigorously evaluated phytochemicals with dual efficacy for both enamel whitening and periodontal biofilm control.

Within this research gap and the rationale with international science references, among various bioactive plant compounds, sesquiterpenes are of particular interest due to their documented antioxidant, antimicrobial, and anti-inflammatory activities. However, the dual-functional application of sesquiterpene-rich extracts for enamel whitening and anti-*P. gingivalis* efficacy remains largely unexplored. Notably, the flower extract of *Magnolia figo* (formerly *Michelia figo*), a traditional botanical used in East Asian medicine, contains a constituent of sesquiterpenes, and its bioactive ingredients have therapeutic potential in disease prevention [20]. Despite its promising pharmacological profile—including apoptosis induction in cancer cells and anti-inflammatory effects [21,22,23,24]—no previous peer-reviewed study has examined *Magnolia figo* flower extract (FMO) as a functional oral care agent. This research gap underscores the rationale of the current study: to provide scientific evidence regarding the functional potential of *Magnolia figo* extract in oral applications in vitro. In particular, we hypothesize that FMO may offer dual benefits: (1) extrinsic enamel whitening and (2) antibacterial activity against periodontal pathogens.

## 2. Results

### 2.1. Effects of FMO on Enamel Shade Recovery

In order to evaluate the whitening efficacy of *Magnolia figo* flower extract (FMO) on the enamel, bovine enamel (*n* = 10, seven experiment groups, a total of 70 pieces of bovine teeth) specimens were treated with varying concentrations of 0.1–3% FMO (treatment group) and compared to coconut oil (vehicle control group) and commercial whitening Colgate toothpaste (positive group). All treatment groups were compared against both an untreated baseline group and the coconut oil to isolate the specific effects of the FMO active compound. Statistical significance was assessed across all groups to ensure that FMO-induced effects were not attributable to the vehicle alone. To evaluate the whitening effects of *Magnolia figo* extract (FMO), colorimetric analysis was conducted on stained bovine enamel specimens (*n* = 10 per group). The color difference (ΔE*) was calculated using the CIELAB color space, where ΔE* > 3.3 and 4.0 represent the perceptibility threshold (PT) and the acceptability threshold (AT), respectively [25,26,27].

**Figure 1:**
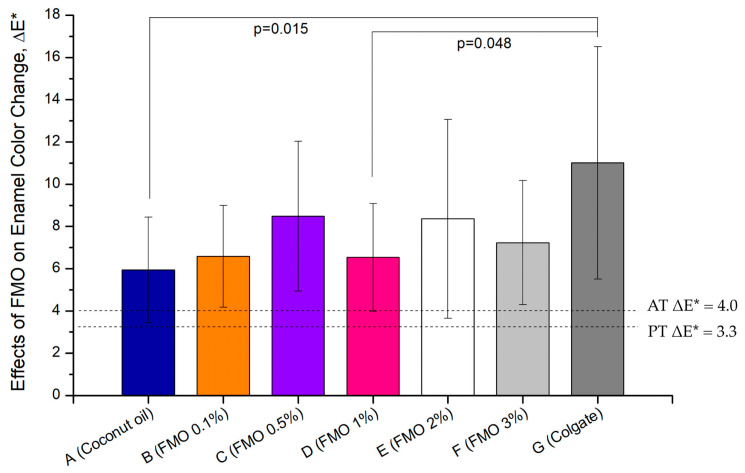
Effects of *Magnolia figo* extract (FMO) on enamel color change (ΔE) following coffee staining and a 7-day treatment: Bovine enamel specimens (*n* = 10 per group) were immersed in coffee solution for 24 h and then treated daily for 7 days with the vehicle control (Group A, coconut oil), FMO at various concentrations (0.1–3%, Groups B–F), and the positive group of the commercial whitening toothpaste (Group G, Colgate). Enamel color change was assessed using the CIELAB color system, with ΔE* calculated to quantify perceptible whitening. Dashed lines indicate the perceptibility threshold (PT, ΔE* = 3.3) and acceptability threshold (AT, ΔE* = 4.0). Data are expressed as the mean ± SD. Statistical comparisons were performed using one-way ANOVA and the Scheff multiple range test. Group G (Colgate) demonstrated the highest overall value of ΔE* = 11.01, showing statistically significant differences when compared to both the vehicle Group A (*p* = 0.015) and the 1% FMO group D (*p* = 0.048). Group B exhibited a value of ΔE* = 6.59 (ΔL = 4.66), and the improvement of shade recovery was modest, suggesting that 0.1% FMO begins to demonstrate efficacy. While Group C exhibited ΔE* = 8.49 (ΔL = 6.71), it showed the most stable and consistent shade recovery performance among all FMO-treated groups. High concentration Groups D, E, and F exhibited greater variability, indicating possible nature-saturated pigment interference at higher doses. These findings reinforce that the observed enamel color recovery is primarily attributable to the bioactive phytochemical compounds present in FMO rather than the carrier oil, as evidenced by the limited effect in Group A.

**Figure 2:**
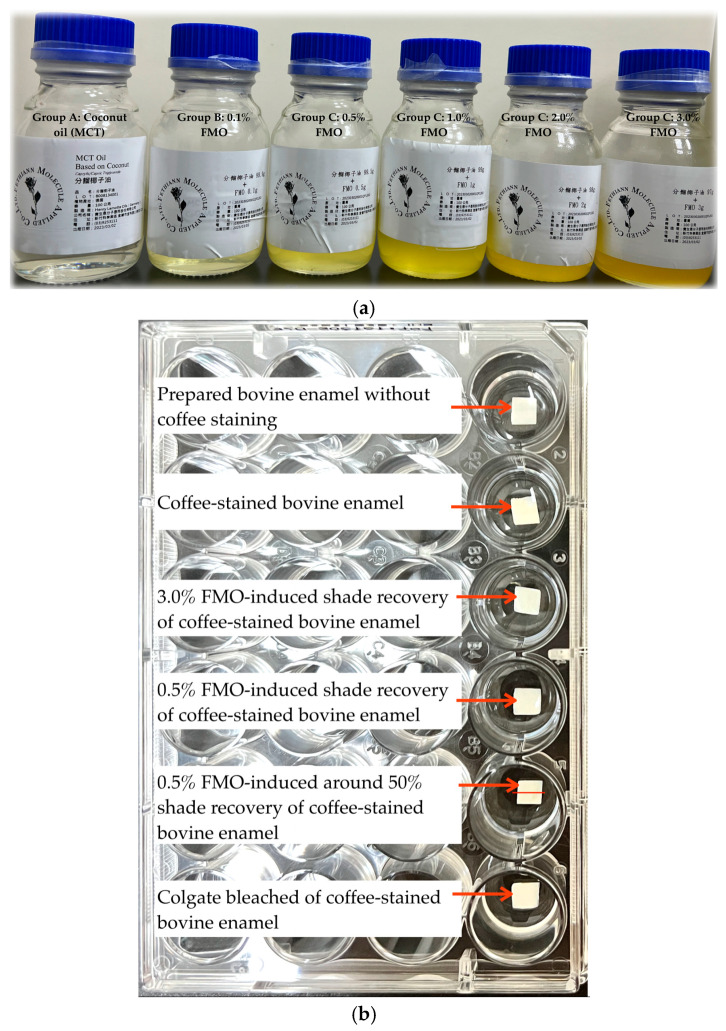
(**a**) The FMO API (active phytochemical ingredient) with different concentrations at 0.1%, 0.5%, 1.0%, 2.0%, and 3.0%. Each one FMO API concentration prepared with coconut oil (“分餾椰子油”) as a carrier oil. (**b**) Representative samples are selectively displayed, including the unstained enamel, the coffee-stained model, the significantly performing treatment group, a representative FMO concentration, and the positive control, in order to highlight the differential whitening responses observed in coffee-stained bovine enamel.

The data shown for the FMO-treated groups exhibited a concentration-dependent increase in ΔE* values compared with the vehicle control group and the positive group (Figure 1, Table 1). Group A (vehicle control–coconut oil) showed a ΔE* value of 5.94 ± 2.50, which is higher than the perceptibility threshold (ΔE* = 3.3) and the acceptability threshold (ΔE* = 4.0), but significantly lower than the FMO and Colgate groups. This effect has almost no obvious performance in enamel shade recovery, making it a valid vehicle control. Group B (0.1% FMO), composed of a low concentration of FMO, achieved a ΔE* value of 6.59 ± 2.41; this suggests that 0.1% FMO induces a perceptible enamel shade recovery effect. Group C (0.5% FMO) possessed the most effective concentration within all FMO-treated groups with a ΔE* value of 8.49 ± 3.55, comparable effect to Group G. It also exhibited lower variance, indicating consistent enamel shade recovery. It represents the optimal balance of whitening efficacy and stability. Group D (1.0% FMO) had a significantly lower value of ΔE* = 6.53 ± 2.56 and ΔL = 4.06 ± 4.01 than Group G (*p* = 0.048), suggesting that increasing FMO concentration beyond 0.5% may not improve whitening and may reduce effectiveness, possibly due to the FMO natural pigment (Figure 2). Group E (2.0% FMO) displayed ΔE* values of 8.36 ± 4.71, indicating shade recovery effectively. Although effective, these data suggest slightly greater variability than Group C, and no statistical difference was observed when compared to Colgate. Group F (3% FMO) displayed ΔE* values of 7.23 ± 2.94, which decreased slightly compared to Group E. This may suggest diminishing returns or saturation compound interactions with FMO natural pigment of high concentrations (Figure 2), which limited efficacy. As expected, Group G (positive control–Colgate) produced the highest value of ΔE* = 11.01 ± 5.50, clearly exceeding the clinical acceptability threshold (AT). However, the large standard deviation indicates inconsistent performance across samples. While effective, the peroxide-based mechanism may explain this variation and raise concerns about enamel tolerance.

### 2.2. FTIR Spectroscopy of FMO Functional Groups and Structures

**Figure 3:**
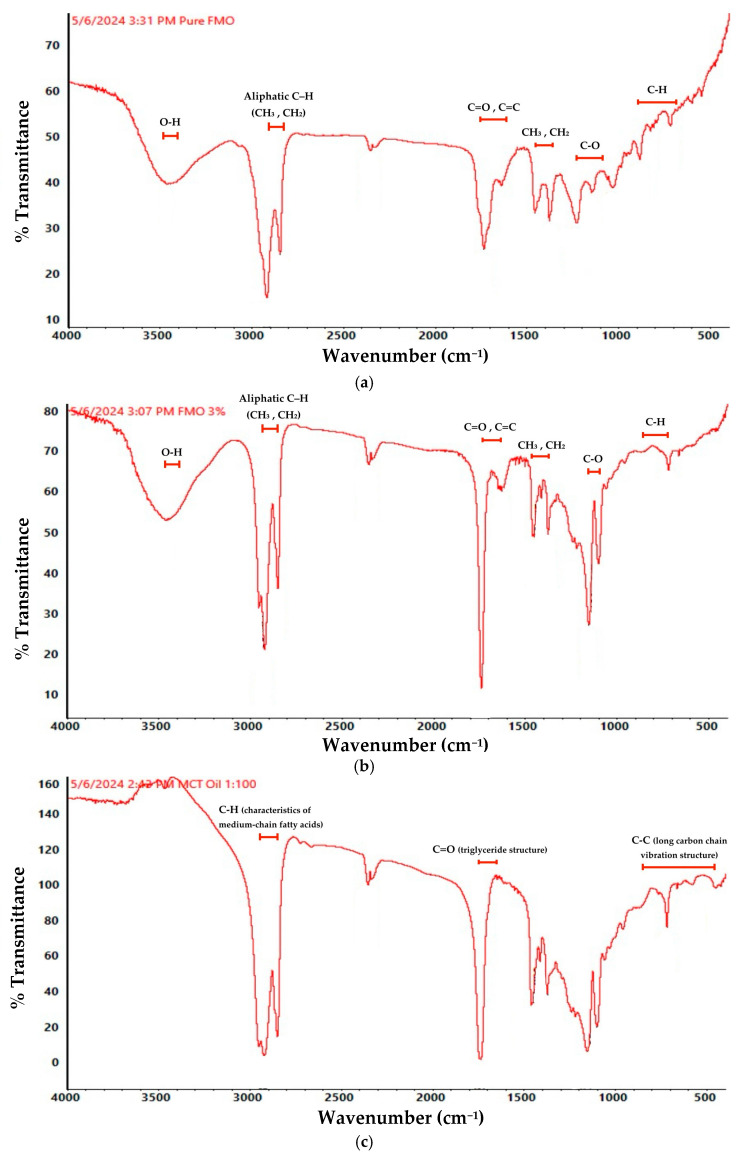
FTIR spectrum of pure FMO (**a**) and 3% FMO in coconut oil (**b**). The complete bioactive functional group combination: the hydroxyl group, the double bond, the ester group, and the ether group; (**c**) the typical structure of long carbon chains and the structure of saturated fatty acids, used only as a vehicle.

FTIR (Fourier-transform infrared spectroscopy) was performed to characterize the chemical functional groups present in the pure FMO extract, the 3% FMO formulation (in which 97% is coconut oil as a carrier), and the coconut oil (MCT: medium-chain triglyceride). The spectra (Figure 3) exhibited clear absorption bands corresponding to key phytochemical constituents (Table 2), notably, those associated with sesquiterpene compounds. Broad O–H stretching vibrations were observed around 3368–3400 cm^−1^, indicating the presence of hydroxyl-containing groups. In the region of 2850–2950 cm^−1^, distinct C–H stretching bands were identified, characteristic of aliphatic –CH_2_ and –CH_3_ groups typically found in long-chain fatty acids and terpenoids. A prominent peak near 1635–1740 cm^−1^ was attributed to C=C stretching vibrations, indicative of the unsaturated sesquiterpene backbone. Additional fingerprint region peaks between 1100 and 1230 cm^−1^ and below 900 cm^−1^ further support the presence of complex terpene derivatives [28,29,30,31]. 

The description in Table 2 details that FTIR spectra of both pure FMO (Figure 3a) and 3% FMO (Figure 3b) dilution revealed consistent peaks corresponding to hydroxyl, alkene, and ether groups, which strongly support the presence of sesquiterpenoids as characterized by GC-MS. In particular, key signals at ~3468 cm^−1^ (–OH), 2950–2850 cm^−1^ (C–H), 1740–1635 cm^−1^ (C=O, C=C), and 1230–1100 cm^−1^ (C–O) match the structural features of major GC-MS-identified compounds such as caryophyllene oxide, spathulenol, and ledene derivatives [21]. This spectral agreement confirms the reproducibility of the sesquiterpene-rich profile of FMO across different analytical methods and validates the phytochemical integrity of the extract used in this study.

### 2.3. Scanning Electron Microscopy (SEM) Analysis of Bovine Tooth with 3% FMO

**Figure 4:**
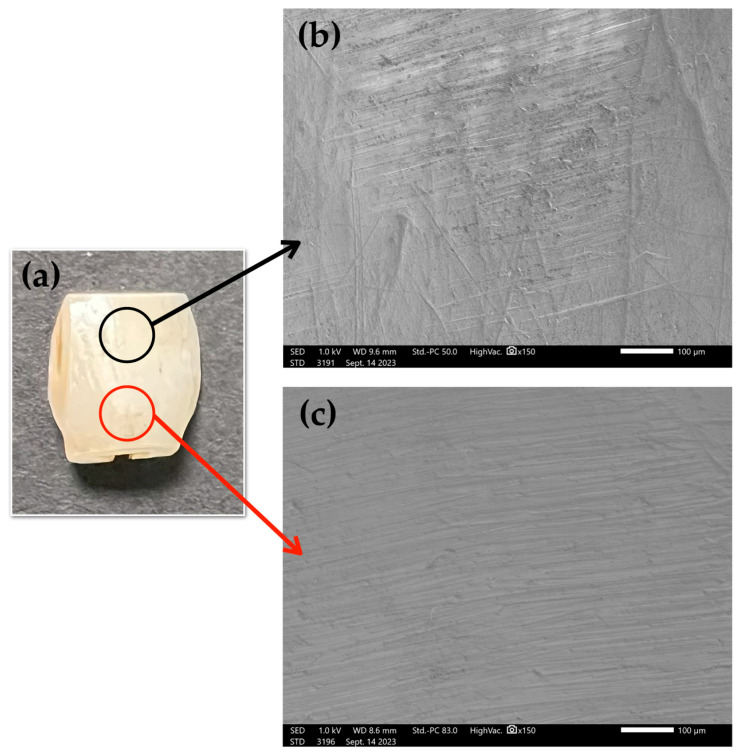
(**a**) A photo of bovine enamel before SEM analysis. SEM analysis was performed to evaluate the effect of 3% FMO treatment on the bovine enamel surface. (**b**) The untreated enamel surface. (**c**) After 12 h immersion in 3% FMO, the enamel surface appeared apparently smoother and structurally intact.

Surface morphology of treated enamel was examined by SEM, which assesses the structural impact of FMO application Figure 4. As shown in the untreated enamel surface (Figure 4b), there are surface scratches and irregular morphology, which may result from mechanical abrasion during sample preparation. In contrast, after 12 h immersion in 3% FMO, the enamel surface appeared apparently smoother and structurally intact (Figure 4c). These results suggest that phytochemical compounds in FMO may interact with the hydroxyapatite matrix via surface chelation or non-covalent bonding, contributing to surface stabilization without demineralization.

### 2.4. Effects of FMO on Porphyromonas gingivalis

To evaluate the dual efficacy of FMO-TCL, both antibacterial and cytotoxicity assays targeting *P. gingivalis* and HGF-1 human gingival fibroblasts, respectively, were conducted.

**Figure 5:**
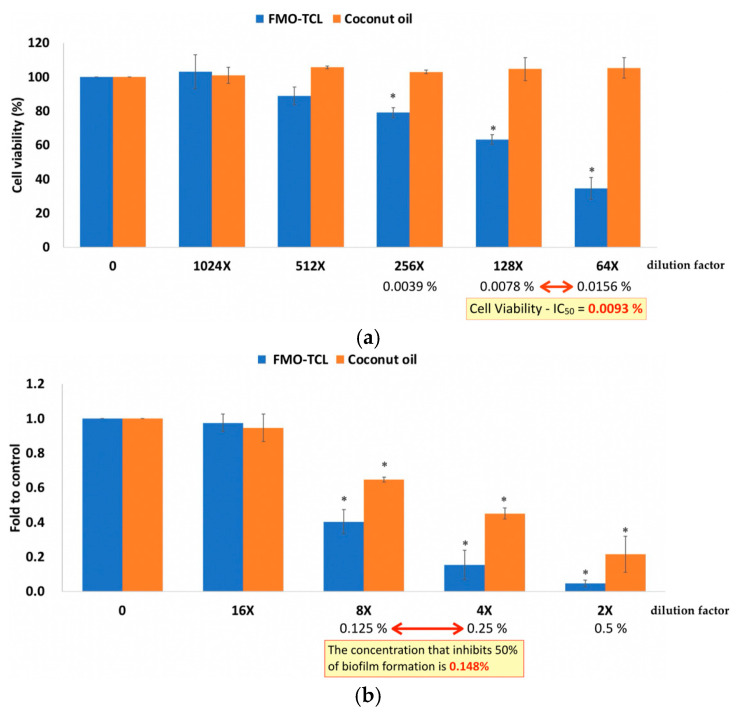
(**a**) Cell viability (MTT) data show that FMO-TCL produced a concentration-dependent reduction in HGF-1 viability. Compared with the control, FMO-TCL at concentrations ≥ 0.0078% (128X) caused a significant decrease in cell viability (* *p* < 0.05), while coconut oil showed no significant cytotoxicity at the tested dilutions. The half-maximal inhibitory concentration (IC_50_) for HGF-1 cytotoxicity was calculated as 0.0093%. (**b**) FMO-TCL significantly inhibited *P. gingivalis* biofilm formation in a concentration-dependent manner; the biofilm inhibition concentration for a 50% reduction (BIC_50_) was 0.148%, indicating efficacy even at low concentrations. Compared with the control, doses of 0.125% (8X) and 0.25% (4X) significantly reduced biofilm formation (* *p* < 0.05), with stronger inhibition observed at a dose of 0.5% (2X) (* *p* < 0.05).

Antibacterial evaluation revealed that FMO-TCL exerted a strong inhibitory effect on the growth and biofilm formation of *P. gingivalis*, a key periodontal pathogen. The minimum inhibitory concentration (MIC) of FMO-TCL was determined to be 0.25%, while the minimum bactericidal concentration (MBC) was 0.5%. In addition, biofilm inhibition analysis using crystal violet staining demonstrated that FMO-TCL significantly suppressed biofilm biomass in a concentration-dependent manner. The biofilm inhibition concentration (BIC_50_)—defined as the concentration required to reduce biofilm formation by 50%—was calculated to be 0.148% (Figure 5b). These results confirm that FMO-TCL is capable of disrupting both planktonic bacterial proliferation and sessile biofilm structures, suggesting its potential utility in the prevention and management of periodontal infections.

To assess the biocompatibility of FMO-TCL on host oral cells, HGF-1 cells were treated with various dilutions of the extract for 24 h, followed by MTT viability assays. As shown in Figure 5, cell viability remained above 80% at concentrations below 0.0078%, whereas reduced viability was observed at higher concentrations. The half-maximal inhibitory concentration (IC_50_) for HGF-1 cytotoxicity was determined to be 0.0093%. This indicates that FMO-TCL retains effective antibacterial and antibiofilm properties at concentrations significantly below its cytotoxic threshold for oral fibroblasts.

### 2.5. Cytocompatibility of FMO

**Figure 6:**
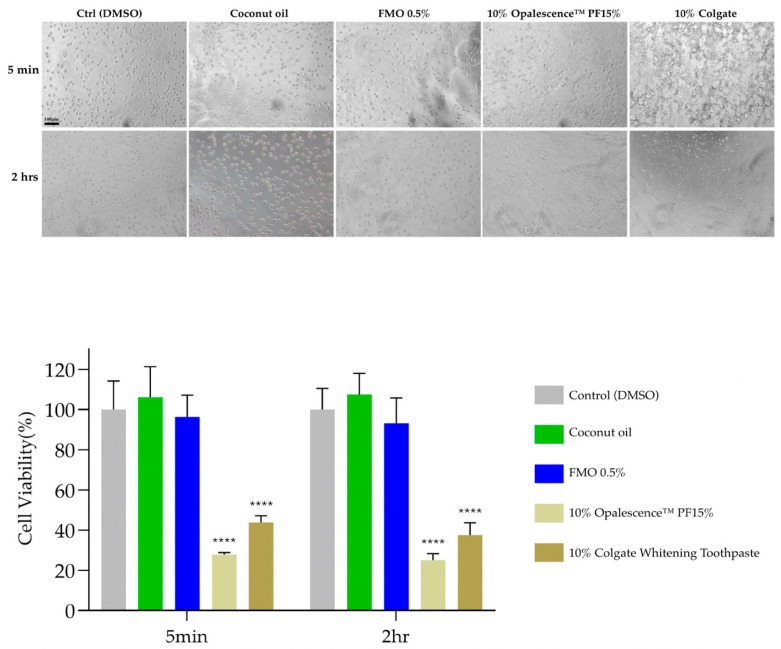
Cell viability of L929 fibroblasts following treatment with varying concentrations of FMO. **** means a significant difference to the control group, *p* < 0.0001. Scale bar: 100 μm.

MTT assays were performed using L929 murine fibroblasts to assess the cytotoxicity of FMO. Cells were exposed to 0.5% FMO, 10% coconut oil, 10% Opalescence PF 15%, and 10% Colgate for 5 min and 2 h. The viability of cells treated with 0.5% FMO and coconut oil remained above 90% at every time point, while the whitening gel and toothpaste significantly reduced viability (**** *p* < 0.0001). These results confirm the excellent cytocompatibility of 0.5% FMO, making them potentially suitable for short- and long-term oral exposure (Figure 6).

## 3. Discussion

This study provides the first comprehensive investigation into the dual-functional potential of *Magnolia figo* flower extract (FMO) in dental applications in vitro, specifically focusing on its enamel-whitening efficacy and antibacterial activity against *Porphyromonas gingivalis*, a key pathogen in periodontal disease. Our findings reveal that FMO not only offers clinically perceptible and acceptable whitening effects on enamel but also exhibits significant inhibitory actions on biofilm formation [11] and planktonic bacteria [12], with excellent cytocompatibility. Although bovine enamel was widely used as a surrogate model for human dental tissues due to its availability and comparable mineral composition, it does not fully replicate the structural and biochemical complexity of human oral environments. However, these results suggest that FMO can be positioned as a novel natural active phytochemical ingredient (API) for promising oral care effects.

In this study, Group G (Colgate) achieved the highest ΔE* (mean ≈ 11), the highest standard deviation, and inconsistent behavior in a* and b* changes, highlighting greater variability. In parallel, FMO demonstrated dose-dependent shade recovery effects on bovine enamel, with ΔE* values apparently exceeding both the perceptibility (ΔE* = 3.3) and acceptability (ΔE* = 4.0) thresholds at concentrations as low as Group B (0.1% FMO). Group C (0.5% FMO) achieved a ΔE* value of 8.49 ± 3.55, indicating robust shade recovery efficacy; these results compare favorably with existing commercial whitening toothpastes. Collectively, these findings confirm that 0.5% FMO achieves comparable whitening efficacy with enhanced color balance restoration and higher stability. Interestingly, Group C (0.5% FMO) often demonstrated superior or comparable performance to Group F (3% FMO), with higher concentration in the VITA shade improvement and pigment removal, particularly with regard to a* (red–green) and b* (blue–yellow) chromatic components. This phenomenon may be attributed to an optimal bioavailability window in which the sesquiterpene compounds and long-chain fatty acid functional groups in FMO can chelate or disrupt surface stains without saturated compound interference from carrier oils.

The structural preservation and enamel compatibility in FTIR and SEM analyses provided mechanistic insights into how FMO interacts with the enamel surface. FTIR spectroscopy confirmed the presence of hydroxyl (–OH), aliphatic (–CH_2_, –CH_3_) and sesquiterpene-associated C=C groups; revealed dominant terpenoid and long-chain fatty acid functional groups; and indicated the potential inclusion β-elemene, γ-elemene, and caryophyllene oxide, consistent (Figure 3a,b, Table 2) with the chemical profile of known anti-inflammatory and anti-microbial plant terpenoids [28,29,30,31]. The *Magnolia figo* flower extract, FMO, as used in this study, is a standardized phytochemical preparation obtained via supercritical fluid extraction, identical in origin and extraction parameters to the sample previously analyzed and published by Chun-Sheng Kuo et al., Molecules, 2023 [21]. In that study, gas chromatography–mass spectrometry (GC-MS) confirmed the presence of multiple sesquiterpenes, including β-caryophyllene, γ-elemene, and caryophyllene oxide [21]. In the present study, FTIR spectra revealed characteristic absorption bands consistent with sesquiterpene structures, such as 3468–3466 cm^−1^, indicating the presence of hydroxyl-containing groups, and 2850–2950 cm^−1^ (alkene C–H stretching), 1635–1740 cm^−1^ (C=C stretching), the 1100–1230 cm^−1^ region, and below 900 cm^−1^ (oxygenated functionalities including C–OH and C–O–C) (Figure 3a,b, Table 2). Although FTIR does not provide compound-level identification, the consistent fingerprint patterns support the presence of sesquiterpenes and complement the previously established GC-MS evidence. Together, these findings form a coherent and reliable analytical chain for the characterization of FMO’s chemical profile in the functional groups.

In light of the observed interactions between FMO’s active phytochemical constituents and the enamel surface, it is scientifically plausible to attribute such effects to the molecular affinity between sesquiterpenes and hydroxyapatite (HA), the primary inorganic component of enamel. HA possesses some lattice rich in calcium ions and surface hydroxyl groups, which are known to engage in non-covalent interactions such as hydrogen bonding and ionic chelation with bioactive plant-derived compounds. Sesquiterpenes, which are lipophilic and often contain oxygenated functionalities (e.g., hydroxyl, epoxide, and carbonyl groups from Figure 3a,b), may facilitate physicochemical interactions with enamel HA, underscoring its exceptional biocompatibility and affinity toward small bioactive molecules. Such interactions not only account for the smoothing effects observed in post-treatment SEM (Figure 4c) but also support the potential of FMO as a natural bio-interactive agent, which may facilitate physicochemical interactions with enamel HA, potentially modifying its surface energy and promoting conformational ordering [32,33,34,35]. The observed peaks suggest possible interactions between sesquiterpene structures and the enamel’s hydroxyapatite matrix through hydrogen bonding or chelation, contributing to surface stabilization. SEM imaging supported this hypothesis by showing that FMO-treated enamel maintained structural integrity and smooth morphology post-treatment. FMO appears to offer an enamel–hydroxyapatite matrix through hydrogen bonding, possibly an adsorption-based mechanism. These surface-preserving effects underscore its potential as a safer alternative to conventional whitening agents.

Antibacterial and anti-biofilm activity against *P. gingivalis* also demonstrate that FMO possesses strong antibacterial effects against *P. gingivalis*, with MIC and MBC values of 0.25% and 0.5%, respectively. Additionally, FMO-TCL significantly inhibited biofilm formation at sub-MIC levels (0.148%), reducing biomass by more than 50%. This dual inhibition of both planktonic cells and sessile biofilm suggests that FMO targets multiple stages of bacterial colonization, which is critical in periodontal pathogenesis. Collectively, these findings highlight the promising therapeutic window of FMO-TCL, which achieves biofilm suppression at concentrations (0.148%) far below its IC_50_ level (0.0093%), thereby supporting its safe and effective application in oral health formulations. Given the increasing interest in the connection between periodontal health and systemic diseases such as Alzheimer’s [15] and cardiovascular conditions [13,14], antimicrobial interventions targeting *P. gingivalis* carry broader implications for public health. While many existing oral antimicrobials fail to penetrate biofilms or present cytotoxicity at bactericidal concentrations, FMO’s low effective concentration and high cytocompatibility provide a compelling therapeutic profile for its potential use.

Cytocompatibility and the safety profile in the MTT cytotoxicity assays on L929 fibroblasts confirmed the excellent cytocompatibility of FMO at 0.5%, with cell viability consistently above 90% even after 5 min and 2 h exposure; 5 min is the time it takes to imitate a human’s daily oral cleaning, and two hours is the approximate time it takes for a “teeth whitening at home” in a dental mouth piece [36]. In contrast, both commercial whitening toothpaste and chemical peroxide gels significantly reduced cell viability (*p* < 0.0001). This suggests that FMO could be safely incorporated into daily-use oral care formulations without the risk of mucosal irritation or systemic absorption concerns, especially for long-term applications.

Although bovine enamel and in vitro biofilm models provide relevant insights into early-stage efficacy and mechanism, they differ in their structural and physiological properties from human enamel. In the future, studies are needed to validate FMO’s effects through in vivo or clinical validation in order to break through limitations and elucidate future perspectives. The dynamic conditions of the oral cavity. including saliva flow, pH fluctuations, and microbiome diversity [37,38], may influence FMO during real-world performance. Additionally, long-term stability, flavor compatibility, and formulation optimization should be explored for product development.

## 4. Materials and Methods

### 4.1. Preparation of Magnolia figo Flower Extract (FMO) and Other Experimental Materials

The *Magnolia figo* flower extract (FMO) was obtained via a patented supercritical CO_2_ fluid extraction process optimized to preserve its sesquiterpenes. The extract was provided by Fethiann Molecule Applied Co., Ltd. (Hsinchu Science Park, Taiwan). The extract was stored in a light-proof container at 20 °C until further use. Dried flower buds were ground and subjected to CO_2_ processing for 8 to 12 h [21]. Afterward, pure FMO was prepated, and working solutions were freshly prepared in coconut oil (MCT: medium-chain triglyceride) as a carrier diluted to the desired concentrations of FMO at 0.1%, 0.5%, 1.0%, 2.0%, and 3.0% (also, it called FMO-TCL) (Figure 2a) for the treatment Groups B to F. Other preparation materials included the following: Group A is the coconut oil for the vehicle control; and Group G is the commercial whitening Colgate toothpaste for the positive group.

### 4.2. Specimen Preparation of Bovine Tooth and Enamel Standardization

Freshly extracted bovine incisors with no stain, cracks, or fractures were collected from a slaughterhouse. All samples (bovine = 70 pieces) were handled under hygienic conditions in accordance with ISO/TR 11405:2015 [39] recommendations for adhesion testing to tooth structure. The preparation procedure was conducted in two main stages: whole tooth processing and enamel specimen preparation [40,41].

Tooth Disinfection and Storage: Bovine incisors were separated from the gingival tissues using a precision diamond wire saw (Tool type: KOSTA DELTA 9 in. BAND SAW) to obtain intact crown segments. Residual soft tissues, including the pulp and connective tissue within the pulp chamber and root canal, were carefully removed using forceps and continuous irrigation under magnification. The teeth were immersed in 75% ethanol for 24 h at room temperature for surface sterilization and disinfection. After disinfection, the specimens were thoroughly rinsed with distilled water and stored in Fusayama/Meyer artificial saliva at 4 °C until preparation of enamel to maintain hydration and simulate intraoral storage conditions.

Enamel Surface Standardization and Slab Preparation: Each bovine incisor was embedded in autopolymerizing acrylic resin with the labial enamel surface exposed. The exposed enamel was sequentially ground using silicon carbide (SiC) abrasive papers of grit sizes #100, #600, and #1200 under water cooling to create a flat and uniform surface. Subsequently, enamel slabs were sectioned using a diamond disc into rectangular specimens with standardized dimensions of approximately 5 mm × 5 mm × 2 mm. To isolate the test surface and prevent lateral dye or treatment penetration, all non-exposed areas of each specimen were sealed with a clear, acid-resistant nail varnish. The exposed enamel surface was further polished using #2000-grit SiC paper under continuous irrigation to produce a smooth, clinically relevant finish. Before testing, specimens were subjected to ultrasonic cleaning in distilled water for 15 min to remove polishing debris and surface contaminants. Finally, the cleaned and polished enamel slabs were stored in fresh artificial saliva at 4 °C to preserve mineral hydration until use in experimental procedures.

### 4.3. Spectrophotometric Color Measurement

The colorimetric evaluation of bovine enamel specimens (*n* = 70) was performed using a calibrated spectrophotometer (VITA Easyshade Advance 4.0, VITA Zahnfabrik, Bad Säckingen, Germany). Prior to each measurement session, the device was auto-calibrated according to the manufacturer’s instructions. Each enamel sample was measured on a standardized and previously marked central region to ensure spatial consistency and repeatability. Three repeated measurements were conducted per specimen, and the average values of L*, a*, and b* coordinates were recorded. Color differences before and after treatment were calculated according to the Commission Internationale de l’Éclairage (CIE) 1976 Lab* color space using the following formula:

ΔE* = √((L1 − L2)^2^ + (a1 − a2)^2^ + (b1 − b2)^2^)

where L*, a*, and b* represent lightness, red–green, and yellow–blue chromaticity coordinates, respectively.

The ΔE* values were plotted and statistically analyzed to assess perceptible and clinically acceptable color differences. Measurements and analytical results are summarized in Figure 1 and Table 1.

### 4.4. FTIR Spectroscopy

ATR Fourier-transform infrared (Attenuated Total Reflection-Fourier Transform Infrared Spectroscopy) spectroscopy was performed using a Thermo Scientific Nicolet Summit LITE spectrometer (Thermo Fisher Scientific, Waltham, MA, USA) equipped with a deuterated triglycine sulfate (DTGS) detector and OMNIC paradigm software (version 2.5) facilities. A total of three samples were prepared, which were pure FMO, FMO 0.5%, and pure coconut oil. These samples were also mixed with the powder of potassium bromide. The sample ratio preparation of 1: potassium bromide (KBr) 5 was prepared in disks. Spectra were recorded in the mid-IR range of 4000–400 cm^−1^ with a spectral resolution of 4 cm^−1^ and 32 scans per sample. Absorption bands related to peaks corresponding to sesquiterpene-associated functional groups (C–H stretch, OH stretch, C=C) were used to validate the presence of key phytochemical structures in Figure 3a,b. The structure of the long carbon chains is shown in Figure 3c.

### 4.5. SEM Observation

Surface morphological changes in bovine enamel (*n* = 1) were analyzed using a JEOL JSM-7800F Prime Schottky Field Emission Scanning Electron Microscope (JEOL Ltd., Tokyo, Japan). One representative *n* =1 was evaluated: Figure 4a shows bovine enamel; Figure 4b presents the surface appearance prior to 3% FMO application, showing visible scratches and micro-defects from mechanical preparation; and Figure 4c presents post-treatment with 3% FMO, showing a smoother enamel surface morphology. This was conducted at 150× magnification and a length of 100 μm under a high vacuum with an accelerating voltage of 1.0 kV. The bovine enamel was coated with platinum for 60 s to reduce electron beam damage.

### 4.6. FMO Verification with HGF and Biofilm Formation Assays on Porphyromonas gingivalis

Human gingival fibroblasts (HGF-1, ScienCell #2630, Carlsbad, CA, USA) were cultured in Fibroblast Medium containing 10% fetal bovine serum (FBS), 1% fibroblast growth supplement, and 1% penicillin–streptomycin. Cells were incubated at 37 °C in a humidified atmosphere with 5% CO_2_. For cytotoxicity assessment, cells were seeded in 96-well plates at 8 × 10^3^ cells/well and allowed to adhere overnight. The following day, cells were treated with FMO-TCL at serial dilutions (0.0039–0.0156%) for 24 h. Cell viability was determined using the MTT assay (Sigma-Aldrich, St. Louis, MO, USA) following standard protocols. The absorbance was measured at 570 nm using a microplate reader (BioTek Epoch 2, Winooski, VT, USA). The half-maximal inhibitory concentration (IC_50_) was calculated by fitting dose–response curves using GraphPad Prism 9.0.

*P. gingivalis* ATCC 33277 was cultured anaerobically in a brain heart infusion (BHI) broth supplemented with 5 µg/mL hemin and 1 µg/mL vitamin K at 37 °C. Bacterial suspensions were adjusted to an optical density of OD_600_ = 0.5 (~1 × 10^8^ CFU/mL) prior to experimentation. The minimum inhibitory concentration (MIC) and minimum bactericidal concentration (MBC) of FMO-TCL were evaluated using standard broth microdilution methods in 96-well plates. The MIC was defined as the lowest concentration inhibiting visible growth after 24 h of anaerobic incubation, while MBC was the lowest concentration showing no viable colonies on BHI agar plates.

To evaluate biofilm inhibition, *P. gingivalis* was seeded in 96-well plates at OD_600_ = 0.5 in the BHI medium treated with FMO-TCL at 0.125%, 0.25%, or 0.5%. After 24 h of anaerobic incubation at 37 °C, wells were washed with PBS, fixed with methanol, and stained with 0.1% crystal violet for 15 min. Excess dye was washed off, and the biofilm-bound dye was solubilized in 30% acetic acid. The absorbance was measured at 590 nm. The concentration inhibiting 50% of biofilm formation (BIC_50_) was calculated based on dose–response curves.

### 4.7. Cell Culture and Cytotoxicity Assay

The mouse fibroblast cell line L929 was cultured in DMEM supplemented with 10% FBS and 1% penicillin–streptomycin at 37 °C with 5% CO_2_. Cells were seeded in 96-well plates at a density of 7000 cells/well and allowed to adhere overnight. The experimental groups (*n* = 3) included the following: 10% coconut oil, 0.5% FMO, 10% whitening gel (Opalescence, 15% PF), and 10% Colgate toothpaste. The exposure durations were 5 min (simulating daily oral care) and 2 h (mimicking home whitening conditions). Post-treatment, the MTT solution (0.5 mg/mL) was added, and cells were incubated for 3 h in the dark. Formazan crystals were dissolved in DMSO, and the absorbance was measured at 570 nm using a microplate reader. Cell viability was calculated relative to the untreated control.

### 4.8. Statistical Analysis

All experiments were performed in triplicate, and the data are presented as the mean ± standard deviation (SD). Statistical analysis was conducted using IBM SPSS (version 25) (SPSS, Inc., Chicago, IL, USA). One-way ANOVA and Scheff multiple range tests were employed to determine significant differences among treatment groups. A *p*-value < 0.05 was considered statistically significant.

## 5. Conclusions

In this in vitro investigation, the *Magnolia figo* flower extract (FMO) demonstrated a dual functional potential as a plant-derived active phytochemical ingredient (API) in oral health applications. The extract exhibited measurable shade recovery effects on stained bovine enamel, as quantified by ΔE* colorimetric analysis. Especially mentioned, ΔE* values represent the sum of deviations on the optical axis, which shift across L*, a*, and b* color axes. As FMO’s active phytochemical ingredients interact with enamel—potentially via chelation—they may alter not only L* (lightness) but also a* (green–red) and b* (blue–yellow) chromaticity, contributing to overall shade recovery. The sesquiterpene of FMO phytochemicals had a significant antibacterial and anti-biofilm effect against Porphyromonas gingivalis, a key periodontal pathogen.

While these findings are promising and suggest that FMO may serve as a candidate for future formulation of natural oral care products, it is important to note that this study was conducted entirely under in vitro conditions using bovine enamel models. Therefore, further in vivo investigations and clinical trials will be essential to validate the efficacy, safety, and potential translational relevance of FMO in human oral environments.

**Figure 7:**
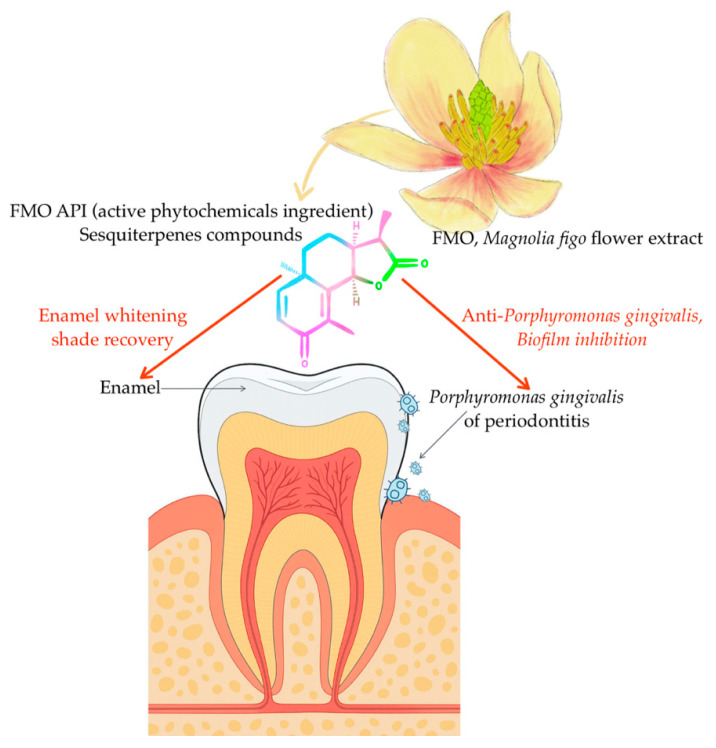
Schematically summarizes the dual-functional potential of *Magnolia figo* flower extract (FMO) based on the present in vitro findings. The whitening efficacy was quantified using ΔE* and VITA shade recovery metrics, while the antibacterial and anti-biofilm effects against *Porphyromonas gingivalis* were confirmed through biofilm inhibition assays. These outcomes provide mechanistic insights into the possible therapeutic applications of sesquiterpene-rich botanicals in oral care from the current study and serve as a mechanistic hypothesis for future in vivo validation.

Overall, this study contributes novel evidence supporting the natural dual functionality of FMO API (Figure 7), offering a foundation for further development and validation in advanced translational models.

## Figures and Tables

**Table 1:** The ΔL, Δa, and Δb were calculated by subtraction of day 7 and day 0 (after stain), expressed as the mean ± SD of the individual 10 samples.

	ΔL	Δa	Δb	ΔE*
A (Coconut oil)	4.66 ± 2.69	−1.10 ± 1.00	−2.64 ± 2.00	5.94 ± 2.50
B (FMO 0.1%)	4.40 ± 2.20	−1.41 ± 0.38	−4.53 ± 1.63	6.59 ± 2.41
C (FMO 0.5%)	6.71 ± 4.64	−2.14 ± 0.77	−2.74 ± 2.69	8.49 ± 3.55
D (FMO 1%)	4.06 ± 4.01	−2.52 ± 1.09	−1.61 ± 2.90	6.53 ± 2.56
E (FMO 2%)	6.24 ± 5.47	−2.09 ± 1.37	−3.09 ± 3.06	8.36 ± 4.71
F (FMO 3%)	4.68 ± 3.69	−1.78 ± 0.79	−4.33 ± 1.96	7.23 ± 2.94
G (Colgate)	8.49 ± 5.39	−2.83 ± 1.27	−5.87 ± 2.66	11.01 ± 5.50

**Table 2:** FMO characteristic absorption using FTIR analysis: phytochemical structure of Figure 3a,b corresponds to GC-MS [21].

FTIR Absorption Wavenumber (cm^−1^)	FTIR Analysis of Indicating Functional Groups/Structural Features (References [28,29,30,31])	Corresponding GC-MS Composition (Reference [21])	Phytochemical Structural Correspondence Description
3468–3400	O–H stretching (hydroxyl)	Spathulenol, Ledene Alcohol, Caryophyllene Oxide	Sesquiterpenols contain hydroxyl (–OH) structures
2950–2850	Aliphatic C–H stretching (aliphatic CH_3_, CH_2_)	β-Elemene, γ-Elemene, Caryophyllene Oxide	All C_15_; sesquiterpenes contain alkyl side chains
1740–1635	C=O, C=C stretching (keto or olefin double bond)	Caryophyllene Oxide, Copaene-8-ol, (1R,3E,7E,11R)-…	Double bond, or oxidation structure, corresponding to epoxy group/olefin structure
1450–1370	CH_3_, CH_2_ bending (methylene)	All sesquiterpenes	Common absorption for aliphatic chains and alkyl side chains
1230–1100	C–O stretching (ether or alcohol)	Spathulenol, Ledene Oxide (II), Ledene Alcohol	Corresponding to the alcohol and ether functional groups in sesquiterpenol
<900	C–H out-of-plane bending (olefins)	β-Elemene, γ-Elemene, Copaene-8-ol	Indicating substituted olefin skeleton structures, such as double bonded benzene rings or cyclohexene structures

## Data Availability

Data is contained within the article.
